# The role of nerve transfers in chronic nerve compression syndromes

**DOI:** 10.1007/s00264-025-06434-2

**Published:** 2025-02-20

**Authors:** Patrick J. Kennedy, Shayoni Nag, Reade Otto-Moudry, Amy M. Moore

**Affiliations:** 1 The Ohio State University College of Medicine, Columbus, OH USA; 2 The Ohio State University Wexner Medical Center, Columbus, OH USA

**Keywords:** Nerve transfer, Compression neuropathy, Supercharge reverse end-to-side

## Abstract

**Purpose:**

Compression neuropathy is a common problem that results in impaired axonal conduction, and with time, numbness, tingling and weakness from muscle atrophy. Supercharge reverse end-to-side (SETS) nerve transfers have emerged as a novel approach to augment function in chronic nerve compression syndromes with minimal donor site morbidity. This review answers the question, “What are the indications, surgical techniques, and nuances of SETS nerve transfers for ulnar, axillary, radial, and femoral compression neuropathies?”.

**Methods:**

This article reviews current literature and technical components of the use of SETS in chronic nerve compression syndromes.

**Results:**

SETS nerve transfers improve functional outcomes and reduce disability in chronic nerve compression syndromes with limited donor site morbidity. SETS nerve transfers for ulnar, axillary, and femoral compressive neuropathy improve muscle strength, as demonstrated by increased MRC scores. It has also been shown that SETS transfers decrease clawing in ulnar nerve compression and pain in axillary nerve compression. More research is needed for SETS transfers for radial nerve compression neuropathies.

**Conclusion:**

SETs nerve transfers have emerged as a novel approach to restore function and reduce pain and dysfunction in chronic nerve compression syndromes. SETS nerve transfers have minimal donor site morbidity and improve the strength and function of muscles innervated by the effected “recipient” nerve. This review explores the indications and surgical techniques of SETS nerve transfers for ulnar, axillary, radial, and femoral compression neuropathies as well as their reported outcomes.

## Introduction

**Fig. 1:**
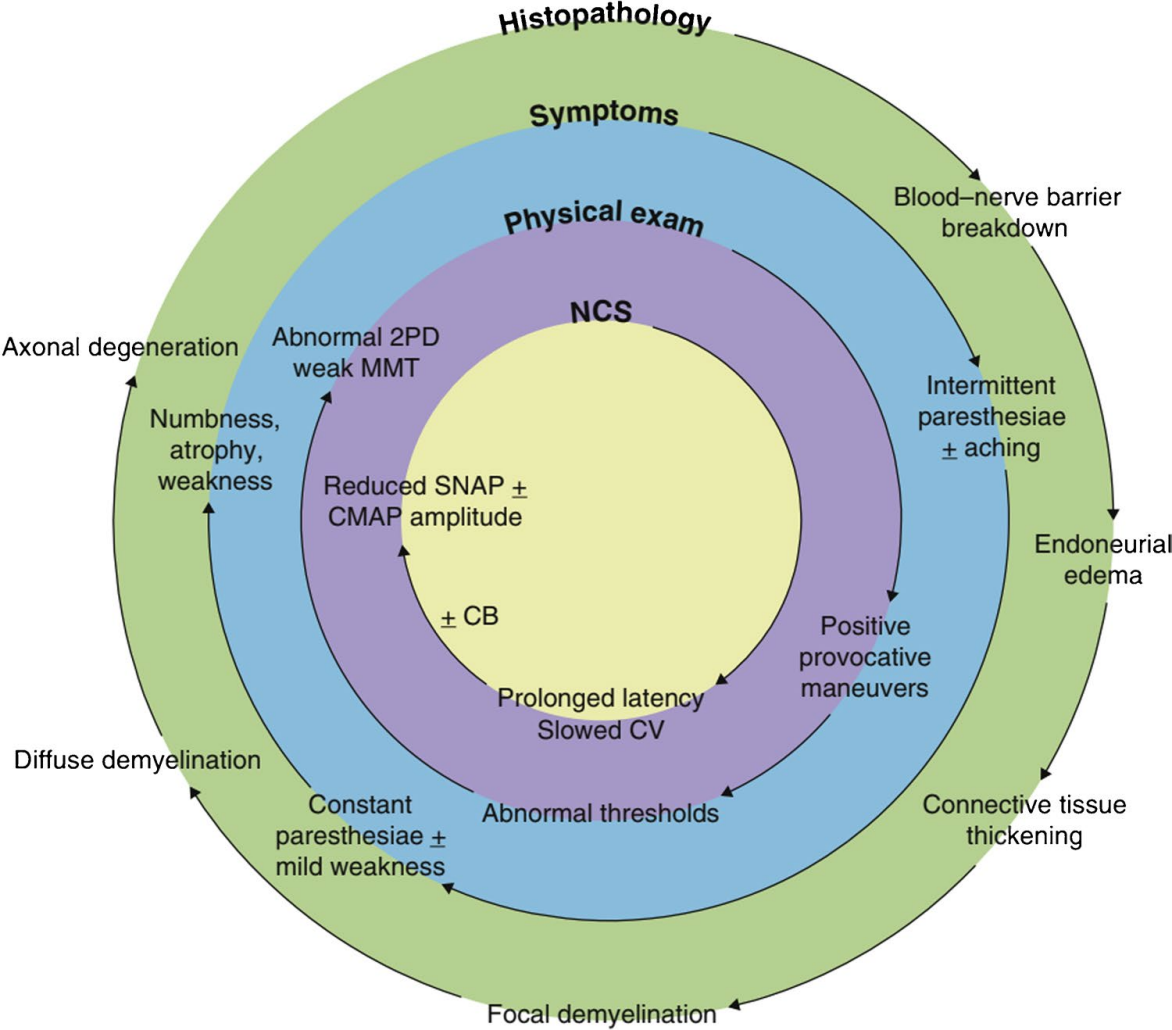
The histopathology of chronic nerve compression and its relationship with symptoms, physical examination findings, and electrodiagnostic results. Abbreviations: 2PD (two-point discrimination), CB (conduction block), CV (conduction velocity), CMAP (compound muscle action potential), MMT (manual muscle testing), NCS (nerve conduction study), SNAP (sensory nerve action potential). Adapted with permission MacKinnon S, in “Nerve Repair,” featured in Nerve Surgery, Thieme, 2015

Compression neuropathy, also known as nerve compression syndrome or nerve entrapment syndrome, is one of the most common nerve injuries seen by hand and nerve surgeons. Although the aetiology of compression neuropathy is multifactorial and debated, the pathophysiology and physical symptomatology are well-established [1]. Chronic compression injuries can cause impaired axonal conduction due to a blood-nerve barrier insult, resulting in axonal degeneration, subperineural oedema, fibrosis, and focal demyelination of the impacted nerve (Fig. 1) [2–4]. The longer the time of compression, the more severe the symptoms including numbness, tingling and eventually weakness from muscle atrophy.

**Fig. 2:**
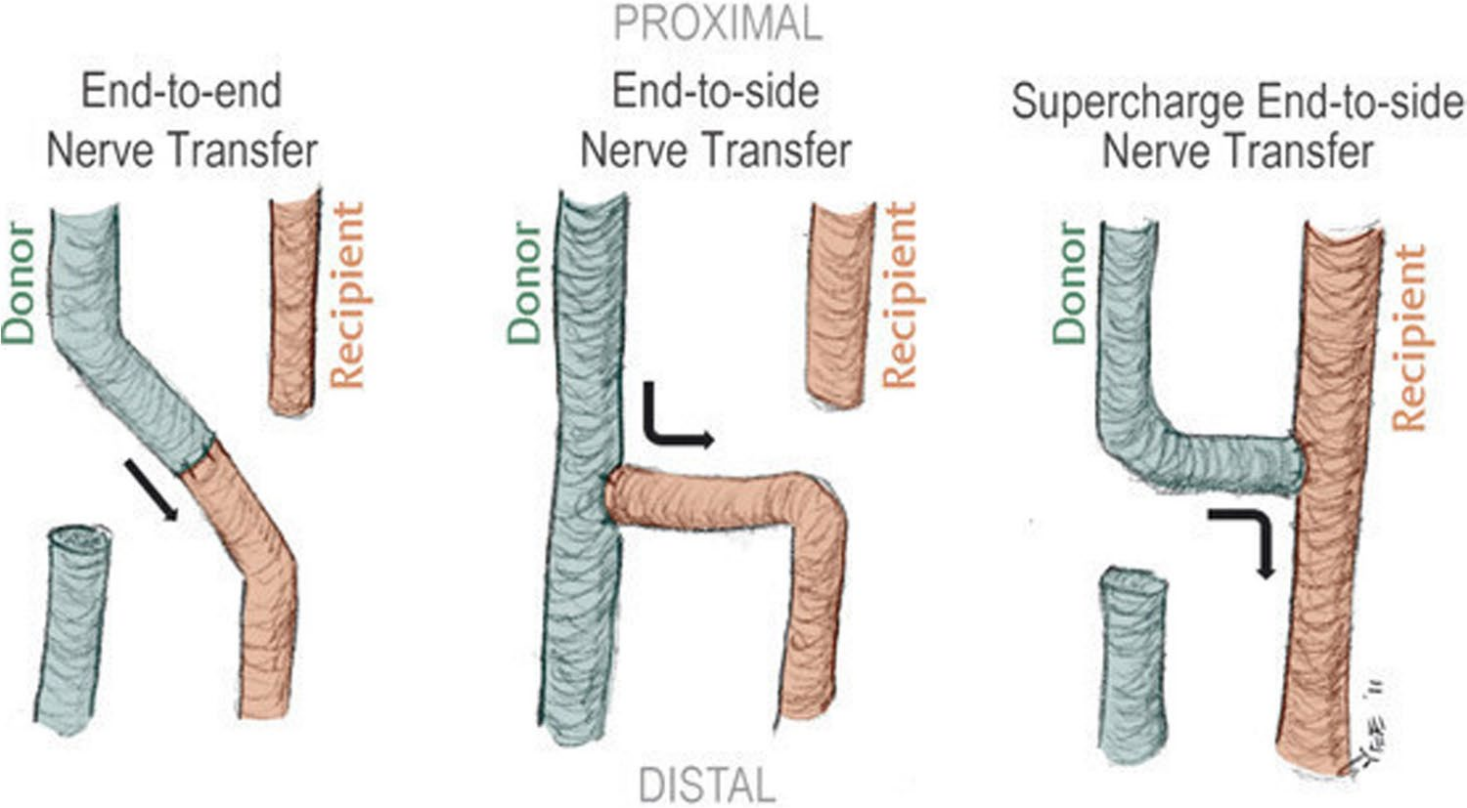
Types of nerve transfers. Primary nerve repairs and nerve grafting are traditionally performed in an end-to-end manner with the native nerve. When a nerve is redirected to another nerve, it is referred to as a nerve transfer. Nerve transfers can involve coaptation in various configurations, including end-to-end, end-to-side, or supercharge end-to-side techniques. Conventional nerve transfers typically connect the donor nerve to the recipient nerve in an end-to-end manner. In an end-to-side nerve transfer, the end of the recipient nerve is attached to the side of the donor nerve. In a supercharge end-to-side nerve transfer, the end of the donor nerve is connected to the side of the recipient nerve. Adapted with permission from Powers H and MacKinnon S, in “Nerve Compression,” featured in Plastic Surgery Principles and Practice, Elsevier, 2022

In chronic compression syndromes, the physical exam and nerve conduction studies can identify the location and severity of the nerve injury. When conservative management fails to relieve symptoms, surgical intervention is warranted. If axonal damage is evident on the electromyogram (i.e. fibrillations) and decreased amplitude is identified on the nerve conduction study, nerve transfer surgery can be considered to augment the recovery of the nerve after decompression. Nerve transfers are surgical procedures that capitalize on the inherent regenerative capacity of the peripheral nervous system to reinnervate a damaged peripheral nerve [4]. There are three types of nerve transfers: end-to-end (ETE, ) end-to-side (ETS, ) and supercharge reverse end-to-side (SETS) (Fig. 2). One of the earliest uses of a SETS was published in 2012, in which the distal anterior interosseus nerve was transferred to the motor branch of the deep ulnar nerve [5]. Studies since have shown that SETS improves intrinsic function, grip and key pinch strength, and DASH Questionnaire scores, making it a useful option for ulnar neuropathies [6–11]. Given the success with the ulnar nerve, the use of SETs nerve transfers has expanded to other sites. The underlying concept of the SETS in compression neuropathy is that the muscle has open muscle endplates, and the nerve transfer performed closer to the target provides an additional source of healthy schwann cells and motor neurons to help restore functional connections to the muscle prior to irreversible atrophy [12, 13]. In this review, indications, surgical details, and nuances of the most common use of nerve transfers in chronic compression syndromes of the extremities are described.

## Indications for SETS in compressive neuropathies

If a patient has weakness, intrinsic atrophy, and electrodiagnostic (EDX) findings of fibrillations in muscles innervated by the suspected compressed nerve on electromyography, the patient is a candidate for SETS in conjunction with surgical decompression. A local, redundant and/or expendable donor nerve is also required. A SETS nerve transfer can augment axonal input connection, potentially increasing the likelihood of recovery [14]. 

### Nerve transfer for ulnar nerve compression

Ulnar nerve compression neuropathy, or cubital tunnel syndrome, is one of the most common compression neuropathies in the upper extremity. Recovery of intrinsic hand function with chronic compression is rare given the distance the injured nerve needs to regenerate [15, 16]. A SETS transfer of the anterior interosseous nerve (AIN) is an option to improve the strength and decrease clawing without causing donor-site morbidity in compression injuries [17–22]. SETS AIN transfers preserve motor endplate and muscle function while also allowing motor function recovery and proximal nerve regeneration in a short time [8]. This transfer is believed to improve recovery of motor hand function as demonstrated by significantly decreased DASH scores [8, 9].

#### Donor nerve

Anterior Interosseous Nerve (terminal branch of median nerve).

#### Adjuvant nerve release location

The authors prefer to perform a Guyon’s canal release distally in the hand to decompress the motor branch as it courses around the hook of the hamate.

### Technical component of surgery

**Fig. 3:**
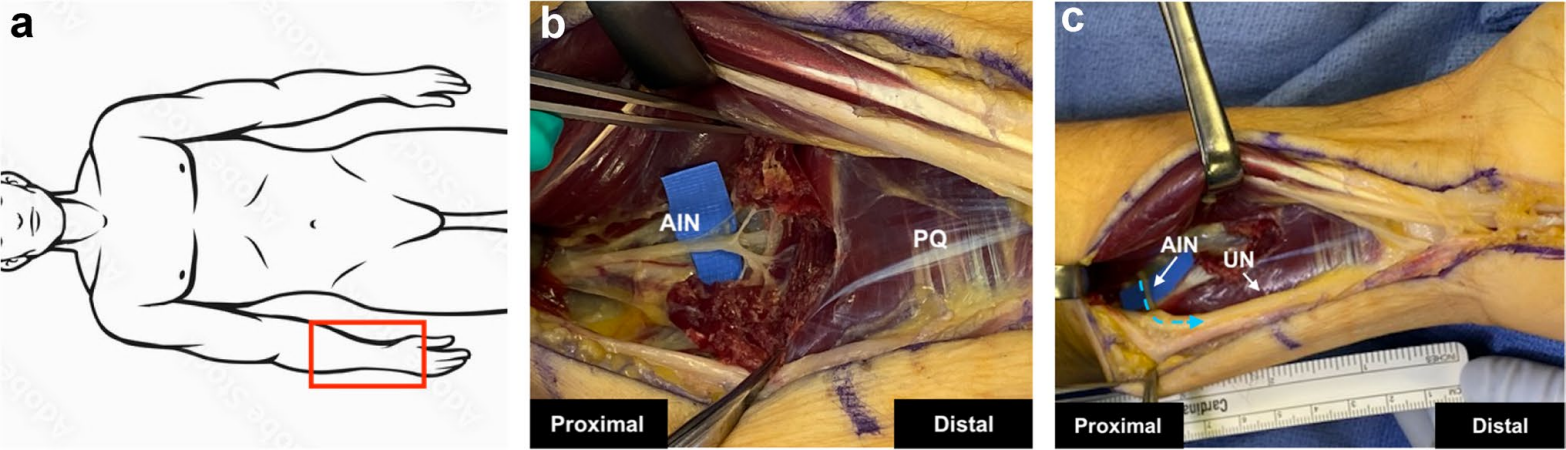
Anterior Interosseous Nerve to Ulnar Motor Supercharge End to Side Transfer (**a**) Red box indicating anatomical orientation of operative field on volar forearm. **b** The anterior interosseous nerve (AIN) is isolated as it branches into the pronator quadratus (PQ). **c** The AIN is coapted through an epineural window created in the motor component of the ulnar nerve (UN). A blue dotted arrow indicates the direction of expected axonal growth

After the Guyon’s canal release, a proximal curvilinear incision is made in the distal forearm extending about 9 cm. Dissection is carried between the flexor mass and ulnar neurovascular bundle. Appropriate understanding of the ulnar nerve topography is essential for the placement of the nerve transfer, but the motor branch does not require physical neurolysis, sparing the vasculature of the nerve and preventing further scarring [4]. Retraction of the superficial and deep forearm finger flexors allows for visualization of the pronator quadratus. The AIN will be found radial to the anterior interosseus vessels at the proximal edge of the pronator quadratus. Using a combination of bipolar cautery and tenotomy scissors, the AIN is neurolysed distally into the muscle until it branches (Fig. 3). The anterior interosseus nerve is transected distally and brought over to the ulnar nerve. The ulnar neurovascular bundle is retracted, and the dorsal cutaneous branch is identified. This branch is followed proximally to the ulnar nerve proper to confirm the topography of sensory–motor–sensory. At this level, about 8–9 cm above the wrist crease, an epineurial window is made over the motor component. The AIN is loosely coapted to cover the two or three fascicles of the motor group of the ulnar nerve. Full range of motion of the wrist and forearm is performed to ensure laxity of the coaptation. To avoid tension, the deep flexors are removed from the ulna for about 3 cm.

## Nerve transfer for axillary nerve Compression

Injury to the axillary nerve is the most common peripheral nerve injury of the shoulder and commonly arises from glenohumeral joint dislocation, humerus fracture, or direct trauma to the deltoid muscle [23]. Common causes of non-traumatic compression injuries to the axillary nerve are quadrilateral space (QS) syndrome and improper crutches use [24, 25]. Compression of the axillary nerve in the QS contributes to ongoing injury, pain, and morbidity [23–25]. In the setting of incomplete nerve injuries, QS decompression with or without a SETS nerve transfer may help. By bringing healthy Schwann cells and additional axons closer to motor endplates, a SETS nerve transfer can be utilized to augment recovering or incomplete axillary nerve injuries [14, 23–25]. End to end nerve transfers have been described for complete axillary nerve injuries [26–28]. However, there are a paucity of studies that specifically analyze the outcomes of SETS in incomplete axillary nerve injury. Chi et al. retrospectively analyzed 23 patients who failed non operative management and were treated with surgical intervention for incomplete axillary nerve injury and/or a nerve transfer [29]. Shoulder abduction strength improved markedly, with 83% of patients achieving functional strength (MRC grade 3 or greater) post-surgery compared to only 17% pre-surgery. Pain scores decreased significantly, and patients reported better overall shoulder function, reflected in improved DASH scores. Further studies analyzing the effects of SETS in incomplete axillary nerve injury are needed [29]. 

### Donor nerve

Branch of the radial nerve to the medial head of the triceps muscle.

### Technical component of surgery

**Fig. 4:**
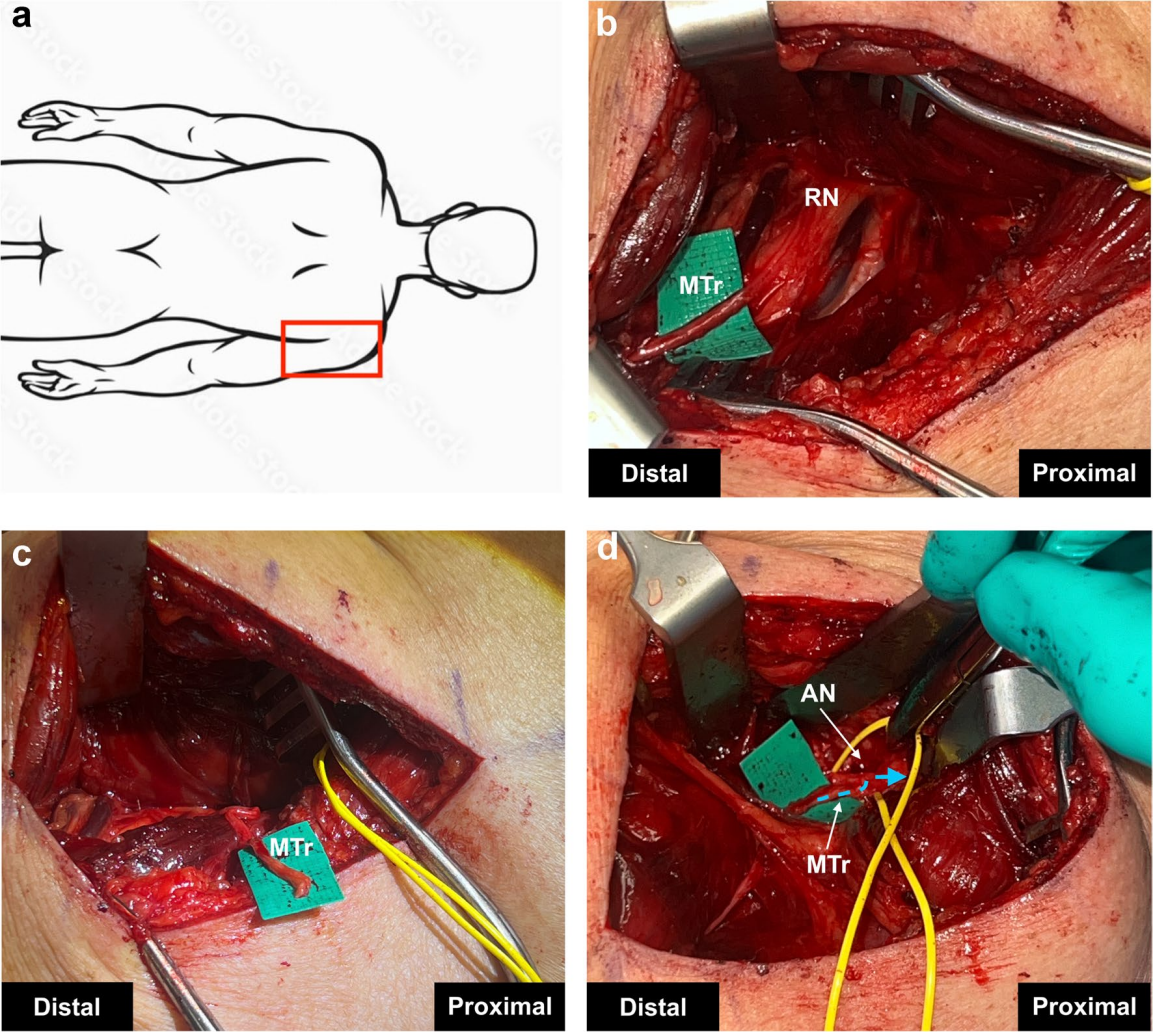
Medial Triceps Branch Nerve to Axillary Nerve Supercharge End to Side Transfer (**a**) Red box indicating anatomical orientation of operative field. **b** The medial triceps branch of the radial nerve (MTr) is identified and isolated from the radial nerve (RN). **c** The MTr is neurolysed, transected distally and transposed towards the axillary nerve (AN). **d** The MTr is coapted through an epineural window to the AN. A blue dotted arrow indicates the direction of expected axonal growth

An incision is made along the free border of the posterior aspect of the arm just distal to the palpable deltoid muscle and is extended distally down towards the olecranon. The subcutaneous tissue is dissected in the interval between the posterior deltoid and the long head of the triceps. The lateral cutaneous nerve branch of the axillary nerve is identified and is followed deeply towards the region of the main trunk in the quadrangular space. The axillary nerve is then neurolysed from any scar tissue proximally, and the anterior, posterior, and teres minor branches of the axillary nerve are neurolysed distally. Handheld stimulation of the axillary nerve is performed. If, after decompression, there is a significant improvement in strength, we do not proceed with the SETS transfer. However, if muscle contraction remains weak after stimulation, the decision to perform a SETS transfer is made. The incision is extended, and the triceps are split. The radial nerve and a branch of the medial triceps muscle is identified, neurolysed and transected distally as it branches in the muscle (to obtain adequate length) (Fig. 4). An epineural window is made in the axillary nerve of the recipient and a tension-free coaptation is performed.

## Nerve transfer for radial nerve Compression

Posterior Interosseous Nerve (PIN) entrapment, i.e. PIN syndrome, is a compressive neuropathy of the PIN that innervates the extensor compartment of the forearm, presenting with finger and thumb extension weakness. It can result from trauma, space-occupying lesions, brachial neuritis, repetitive pronation/supination, and spontaneous compression [30, 31]. Certain anatomical variants, including the radial nerve passing through the Arcade of Frohse to become the PIN, the radial recurrent vessels, and tendinous proximal border of the extensor carpi radialis brevis can cause impingement [32]. Decompression of the posterior interosseous nerve involves releasing these compressive structures. There is a paucity of literature on the use of a SETS transfer in this setting, but applying the concepts of a SETS, the supinator branches are expendable to transfer to the PIN if the musculocutaneous nerve and biceps muscle function with strong supination is intact.

### Donor nerve

Nerve to Supinator (Branch of the Radial Nerve).

### Technical component of surgery

**Fig. 5:**
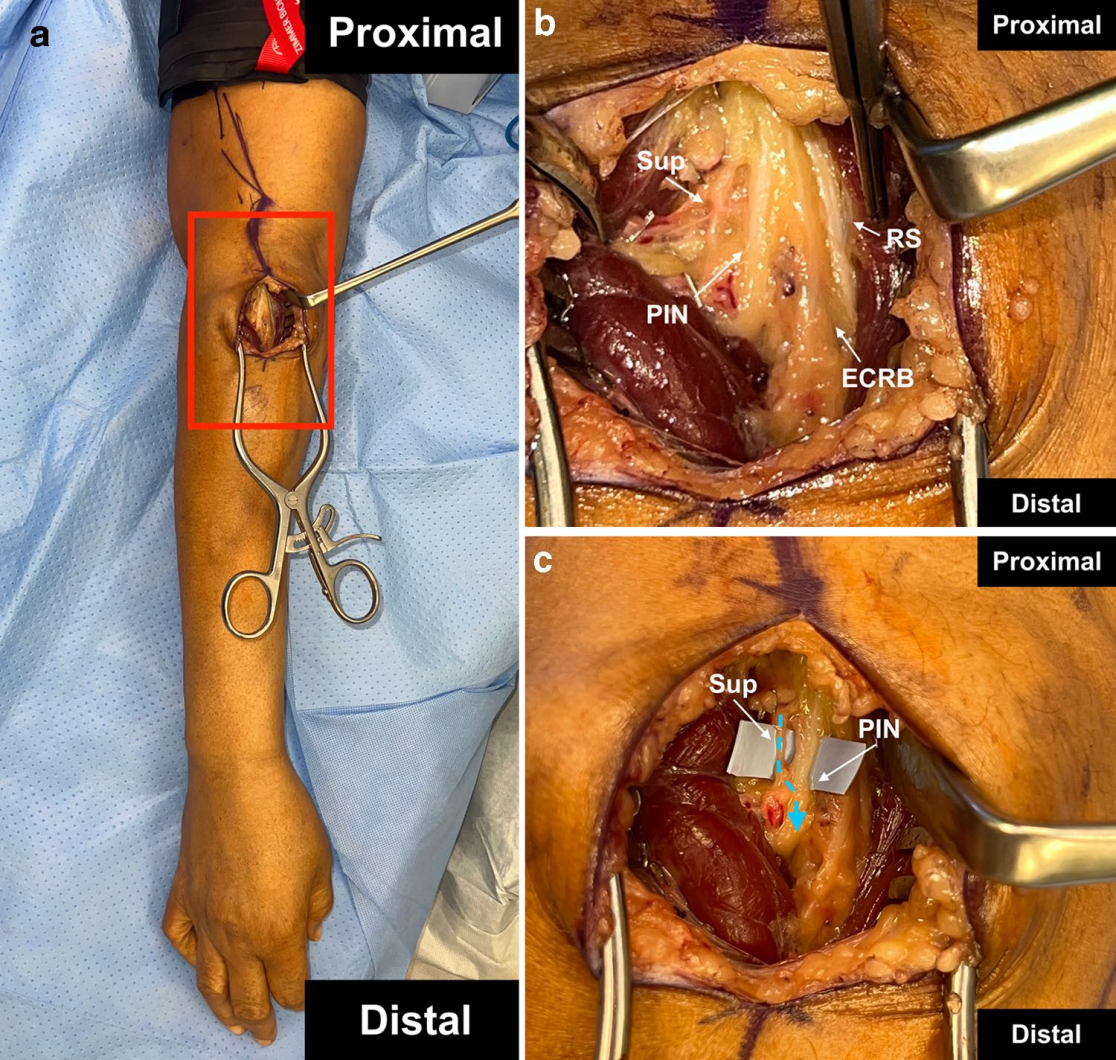
Supinator Nerve to Posterior Interosseous Nerve Supercharge End to Side Transfer (**a**) Red box indicating anatomical orientation of operative field. **b** A branch of the radial nerve to the supinator muscle (Sup), the posterior interosseous nerve (PIN), the extensor carpi radialis brevis (ECRB), and the radial sensory (RS) branch are identified. **c** The Sup is coapted through an epineural window to the PIN. A blue dotted arrow indicates the direction of expected axonal growth

An incision is made just lateral to the palpable brachioradialis (BR) muscle. The subcutaneous tissue is dissected, and the interval between the BR and extensor carpi radialis longus (ECRL) muscle bellies are identified. The muscle interval is dissected, and the radial sensory nerve branch is found at the base of the muscle. Any crossing vessels that compress nerve branches are ligated. The radial sensory nerve is followed proximally and the extensor carpi radialis brevis (ECRB) nerve and PIN are identified proximally. The compressive ECRB tendinous fascia is elevated and released sharply with scissors. The supinator muscle is then exposed and its fascia is transected. Next, the nerve(s) to the supinator are identified and stimulated. The PIN is then stimulated, and if there is weak stimulation an end-to-side supinator SETS transfer to the PIN may be performed. The supinator branches are then transected distally and transferred through an epineurial window to the PIN with a tension free coaptation (Fig. 5).

## Nerve transfer for femoral nerve compression

Lower extremity nerve compression can be a result of a variety of aetiologies, including benign irritation, kneeling, surgical positioning, tumours, and retroperitoneal haemorrhage. Symptoms include sensory loss, weakness, pain, numbness and tingling, loss of coordination, and ambulation difficulties. Femoral nerve palsies are most commonly caused during orthopaedic, intrabdominal, vascular, or gynecologic operations [25, 29]. The site of compression can be retroperitoneally or as the nerve passes under the inguinal ligament. These injuries can cause weakness in knee extension, hip flexion and sensory loss of most of the anterior lower extremity [33]. Two-thirds of patients with a femoral nerve compression injury experience functional improvement within two years of symptoms, but many have residual symptoms [34]. Both the sartorius motor nerve and obturator nerve can be used as donors for nerve transfer in the setting of femoral nerve compression. McInnes et al. found that the sartorius branches are ideal donors due to their proximity and adequate supply of nerve fibres with each branch containing an average of 672 nerve fibers [35]. In this study, the majority of patients underwent SETS nerve transfers, with only one undergoing ETE, for femoral neuropathy and all demonstrated an MRC score of greater than or equal to 4-/5 for knee extension and with significantly improved pain scores [35]. In the setting of severe weakness, it is preferred to use as many donors as possible: obturator nerve branches to gracilis and adductor longus muscles, as well as, the sartorius nerve branches. If all nerve branches are weak, then the priority recipient nerves are the rectus femoris and vastus medialis to stabilize the medial and anterior knee as the lateral knee is often stabilized through the tensor fascia latae muscle which is innervated by the inferior gluteal nerve.

### Donor nerve

Sartorius Motor Nerve (Branches of the Femoral Nerve) and/or Obturator Nerve (Branches of the Femoral Nerve).

### Technical component of surgery

**Fig. 6:**
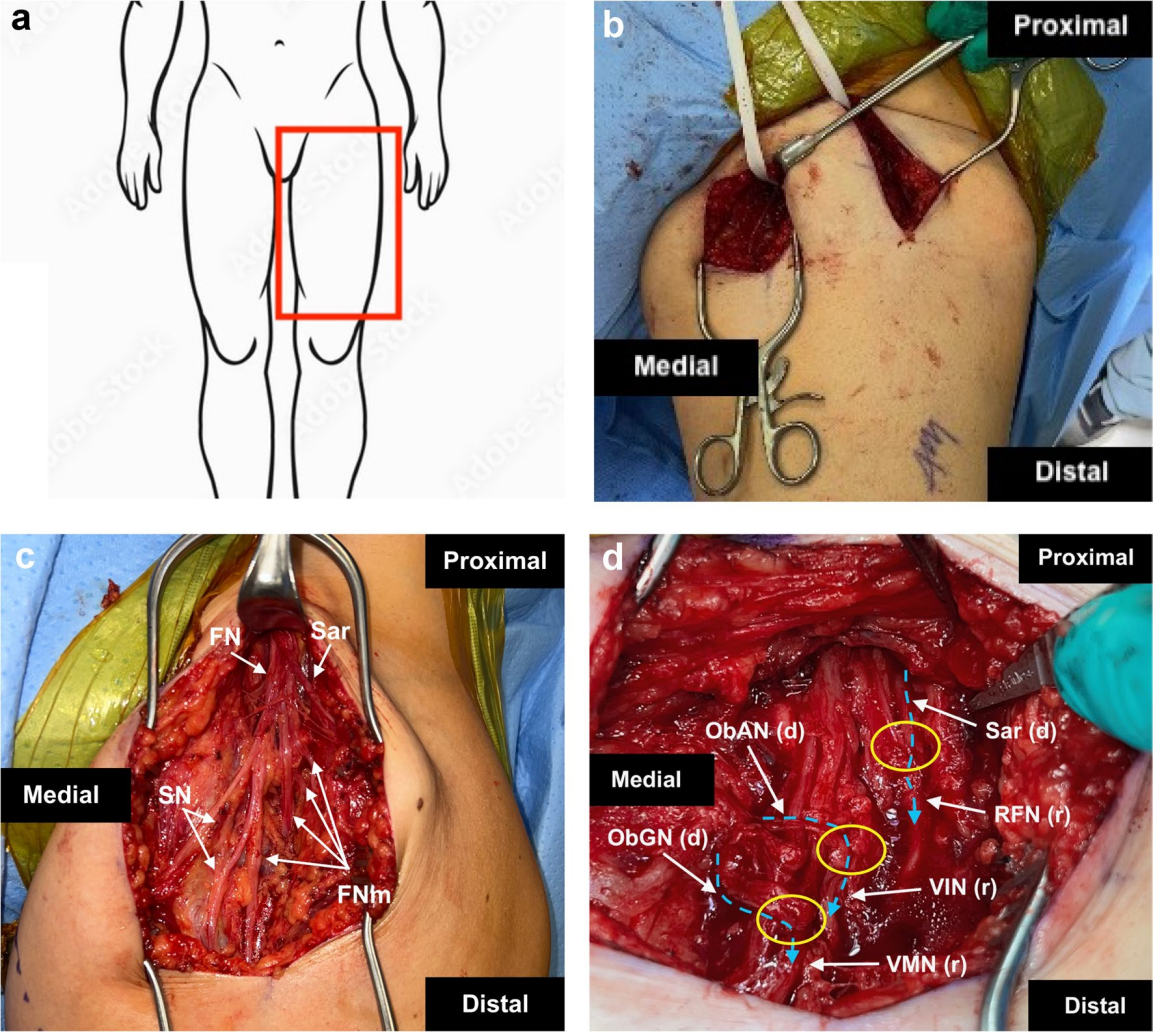
Sartorius and Obturator Nerves to Femoral Nerve Supercharge End to Side Transfer (**a**) Red box indicating anatomical orientation of operative field. **b** A Penrose drain marks the tunnel between the incisions of the obturator nerve exposure and the femoral nerve exposure. **c** The branches of the femoral nerve (FN) are isolated. The motor branches of the femoral nerve (FNm) are identified lateral to the saphenous nerve (SN). **d** The obturator nerve branches to the gracilis (ObGN), adductor longus muscles (ObAN), and the branch of the femoral nerve to the sartorius (Sar) serve as donors **d** and are transferred to the side of the recipient (r) FNM branches innervating the vastus medialis (FMN), vastus intermedius (VIN), and rectus femoris (RFN) respectively. Coaptations are indicated by yellow circles. A blue dotted arrow indicates the direction of expected axonal growth

A longitudinal incision is made below the inguinal crease, lateral to the palpable femoral pulse. Three layers of fascia are encountered during dissection down to the femoral nerve. First, the superficial fascia is incised, followed by the second fascia which covers the sartorius muscle. Deep to the second layer lie the sensory and superficial motor branches of the femoral nerve, which innervate the sartorius muscle. These nerves should be preserved as they are often spared in femoral nerve injuries and, if functional, can be used for nerve transfer. After the sartorius branches are identified, the medial border of the sartorius muscle is retracted and dissection reveals the quadriceps branches beneath the femoral sheath, the third fascial layer. The saphenous portion of the femoral nerve is the most medial branch, then (from medial to lateral) are the branches to the vastus medialis, vastus intermedius, vastus lateralis, and rectus femoris muscles (lying more superficial) (Fig. 6).

The quadriceps branches are stimulated to assess function. Depending on severity of weakness and presence or lack of a sartorius muscle function, a SETS transfer can be performed. In cases where the obturator nerve is used, a second longitudinal incision is made below the palpable adductor longus tendon. The interval between the adductor longus and gracilis muscles are bluntly dissected, revealing the neurovascular bundle to the gracilis muscle. The obturator nerve branch to the gracilis is identified and neurolysed proximally and additional motor branch to the adductor longus is also encountered. Both branches have enough length to transfer anteriorly to the femoral nerve. After confirmation via electrical stimulation, the branches are mobilized and transected as distally as possible (preferably to the point of entrance into the muscle) to preserve length. A tunnel is created to connect the anterior and medial thigh incisions for nerve transfer. The posterior branch of the obturator nerve is preserved to maintain adductor magnus function and thigh adduction. The donors are transposed to the femoral motor branches and nerve coaptation through epineurial windows are performed. The leg is moved through its full range of motion to ensure a tension-free nerve repair.

## Conclusion

The use of SETS nerve transfers for chronic compression remains controversial but feasible with little, if any, morbidity. These transfers use the inherent regenerative ability of peripheral nerves to restore function by redirecting neural connectivity, providing a source of healthy Schwann cells and additional motor neurons. Successful nerve transfer surgeries rely on careful donor nerve selection, neurolysis, and tension-free coaptations. In patients with severe nerve compression injuries, nerve transfers should be considered to augment decompression surgeries with the goal to improve muscle strength, restore vital movement and minimize disability.

## Data Availability

No datasets were generated or analysed during the current study.
